# Association of early childhood caries and malocclusion patterns: A pedodontic-orthodontic perspective

**DOI:** 10.6026/973206300223412

**Published:** 2026-06-30

**Authors:** Jnana Ranjan Swain, Chilukuri Raghuram, Naveena Vyshnavi K., Sugandha Sharma, Lata Kumari, Anushi Mehandiratta

**Affiliations:** 1 Department of Pediatrics and Preventive Dentistry, Institute of Dental Sciences, Siksha O Anusandhan (Deemed to be) University, Bhubaneswar, Odisha, India; 2 Department of Pediatric and Preventive Dentistry, Practitioner at Shirdi Sai Multi Speciality Dental Hospital, Hyderabad, Telangana, India; 3 Department of Pediatric and Preventive Dentistry, Government dental college, Vijayawada Andhra Pradesh, India; 4 Department of Pediatric and Preventive Dentistry, Consultant, Lather Dental Clinic and Implant Centre, Bhiwani, Haryana, India; 5 Department of Pediatric and Preventive Dentistry, SGT Dental College and Research Institute, Gurugram, Haryana, India

**Keywords:** Early childhood caries (ECC), malocclusion, pediatric dentistry, occlusal development, risk factors

## Abstract

Early childhood caries (ECC) is a major pediatric oral health problem that may adversely affect normal occlusal and craniofacial development in children. Therefore, it is of interest to evaluate the relationship between early childhood caries (ECC) and developing malocclusion patterns in young children. A cross-sectional pedodontic-orthodontic assessment was conducted to determine how caries severity influences occlusal development. Data show a significant association between severe ECC and malocclusions such as increased overjet, crossbite and anterior open bite. Behavioral and dietary factors further contributed to this relationship. Early preventive intervention and integrated dental care are essential to reduce long-term developmental issues.

## Background:

Early childhood caries (ECC) is recognized as one of the most common chronic diseases affecting children worldwide, posing a significant burden on oral and general health. It is defined as the presence of one or more decayed, missing, or filled tooth surfaces in any primary tooth in children under six years of age [1]. ECC is a multifactorial disease with a complex etiology involving cariogenic microorganisms, frequent consumption of fermentable carbohydrates, inadequate oral hygiene practices and various social and environmental determinants [4]. The high prevalence of ECC across both developing and developed nations highlights its importance as a major public health concern. Beyond its immediate consequences such as pain, infection and difficulty in eating and speaking, ECC has long-term implications on the growth and development of the craniofacial complex. If left untreated, ECC can progress rapidly, often leading to extensive destruction of tooth structure and eventual premature loss of primary teeth [5]. The premature loss of primary teeth is a critical factor influencing the development of occlusion. Primary teeth play a vital role in maintaining arch length, guiding the eruption of permanent successors and preserving the balance of occlusal forces within the dental arches. Loss of these teeth at an early age disrupts this balance, resulting in mesial migration of adjacent teeth, reduction in arch space and increased risk of crowding and malalignment [6]. Furthermore, several studies have demonstrated a significant association between ECC and the development of malocclusion. Children affected by ECC are more likely to exhibit occlusal discrepancies such as altered molar relationships, loss of primate spaces and crowding in the developing dentition [7]. Changes in occlusal characteristics, including spacing patterns and arch relationships, have also been reported in children with a history of severe caries experience [8]. In addition to structural changes, ECC can also influence functional aspects of the stomatognathic system. Pain and discomfort associated with carious lesions may lead to altered masticatory patterns, reduced chewing efficiency and asymmetrical muscle function. These functional disturbances may adversely affect jaw growth and contribute to the development of malocclusions such as crossbite and abnormal overjet [3]. Behavioral factors further strengthen the link between ECC and malocclusion. Prolonged bottle feeding, nocturnal feeding practices and non-nutritive sucking habits such as thumb sucking and pacifier use are commonly associated with ECC. These habits can exert abnormal forces on developing dental arches, leading to occlusal anomalies like anterior open bite, increased overjet and posterior crossbite [9]. Therefore, ECC should not be viewed solely as a disease of dental hard tissues but as a condition with broader implications on occlusal development and craniofacial growth.

## Methodology:

The present study employs a cross-sectional observational methodology to investigate the association between early childhood caries (ECC) and malocclusion patterns from a combined pedodontic-orthodontic perspective. The study population will consist of pre-school children aged 3 to 6 years, selected through stratified random sampling from multiple sources including local pre-schools, anganwadi centers, community health camps and pediatric dental clinics to ensure diversity and representative distribution across age, gender and socio-economic backgrounds. Eligibility criteria will include healthy children within the specified age range who have not received prior orthodontic therapy, have no history of systemic disease, craniofacial anomalies, or developmental syndromes that could influence dentofacial growth. Written informed consent will be obtained from parents or guardians prior to participation. All clinical examinations will be carried out by two trained and calibrated examiners a pedodontist and an orthodontist to ensure consistency and inter-examiner reliability. Calibration exercises will be performed on 10% of the sample before data collection to minimize diagnostic variability. Examinations will be conducted in a well-lit environment using disposable mouth mirrors, probes and protective equipment in accordance with infection control protocols. ECC will be assessed using WHO criteria and severity will be documented through both DMFT and DMFS indices to record decayed, missing and filled teeth or surfaces. Caries activity, presence of white spot lesions and patterns of decay (smooth surface, pit and fissure, or rampant caries) will also be noted. The assessment of malocclusion will be performed using standard orthodontic diagnostic parameters. These will include evaluation of molar and canine relationships according to Angle's classification, measurement of overjet and overbite using a calibrated ruler, documentation of crossbite (anterior or posterior), scissor bite, open bite, deep bite, dental crowding, spacing, midline deviation and any functional shifts. Clinicians will also observe oral habits such as thumb sucking, tongue thrusting and mouth breathing, as these may contribute to malocclusion and act as confounding variables when interpreting the association with ECC. A structured and validated questionnaire will be provided to parents or guardians to record demographic characteristics, socioeconomic indicators, feeding practices (breastfeeding, bottle-feeding, night feeding), dietary patterns, frequency of sugar exposure, oral hygiene habits, parental supervision during brushing, fluoride exposure and dental attendance history. These variables will help identify behavioral and environmental factors that may influence both ECC and malocclusion development. All collected data will be entered into an encrypted database and cross-checked for accuracy by an independent reviewer. Statistical analysis will be performed using SPSS software. Descriptive statistics will summarize ECC prevalence, malocclusion distribution and demographic variables. Inferential tests such as chi-square, independent t-tests and logistic regression will be used to determine significant associations between ECC (presence and severity) and specific malocclusion traits while adjusting for confounders. Odds ratios with 95% confidence intervals will be calculated to quantify risk relationships. A p-value < 0.05 will be considered statistically significant. Ethical clearance will be obtained from the institutional ethics review board prior to study initiation. All procedures will comply with the Declaration of Helsinki. Children identified with urgent dental needs during the study will be referred for appropriate treatment at no cost to ensure ethical responsibility and community welfare.

## Results:

A total of 240 children aged 3-6 years participated in the study, comprising 122 boys (50.8%) and 118 girls (49.2%). The overall prevalence of Early Childhood Caries (ECC) was found to be 61.7%, with a mean dmft score of 3.42 ± 1.8. Table 1 presents the distribution of ECC severity categories across the study sample. Children with severe ECC (S-ECC) represented 28.3% of all cases, indicating a notable burden of untreated dental decay in preschoolers (Table 1).Malocclusion traits were identified in 72.5% of the total children examined. The most frequently observed malocclusion patterns included increased overjet (22.1%), anterior open bite (17.5%), anterior crossbite (11.7%), posterior crossbite (8.8%) and mild-moderate crowding (18.3%). Angle's molar classification showed that 68.7% had Class I, 21.6% had Class II and 9.7% had Class III molar relationships. A significantly higher occurrence of malocclusion was recorded among children presenting with ECC compared to caries-free children (p < 0.05).On comparing ECC status with malocclusion patterns, a statistically significant relationship was observed between ECC and anterior open bite (p = 0.011), increased overjet (p = 0.017) and anterior crossbite (p = 0.023). As shown in Table 2, 31.6% of children with severe ECC exhibited increased overjet compared to only 9.5% in the caries-free group. Similarly, anterior open bite was present in 24.2% of the S-ECC group, whereas it was seen in only 8.6% of children without ECC. Logistic regression confirmed that children with severe ECC were 2.4 times more likely to exhibit anterior open bite and 1.9 times more likely to present with increased overjet after adjusting for feeding habits and oral hygiene practices. Furthermore, functional habits such as prolonged bottle-feeding and non-nutritive sucking were significantly associated with both ECC and malocclusion occurrence (p < 0.05), suggesting that these behaviors may contribute simultaneously to dental decay and developing occlusal discrepancies. However, no significant association was observed between ECC and molar relationship classifications (p > 0.05), indicating that skeletal malocclusion patterns may be less influenced by caries activity in early childhood. The results collectively reveal a clear pattern wherein the severity of ECC correlates strongly with the presence of detrimental malocclusion traits. These findings emphasize the need for integrated pedodontic-orthodontic screening during early childhood to prevent long-term dental complications.

## Discussion:

The findings of the present study align with an extensive body of literature emphasizing the interconnected nature of ECC, malocclusion and various behavioral and environmental determinants. The association between early childhood caries (ECC) and malocclusion is multifactorial, involving biological, behavioral and socioeconomic determinants. The present discussion highlights how these interconnected factors contribute to altered occlusal development in children affected by ECC. Dietary and feeding practices play a central role in the development of ECC and indirectly influence malocclusion. Frequent consumption of sugary foods, prolonged bottle feeding and nocturnal feeding habits create a cariogenic environment, increasing the risk of ECC [10]. These same practices are often associated with prolonged non-nutritive sucking habits, which can alter the equilibrium of orofacial muscles and lead to malocclusions such as anterior open bite, increased overjet and posterior crossbite [18]. Preventive guidelines emphasize the importance of early intervention in reducing ECC and its long-term consequences. Recommendations by pediatric dental authorities stress early oral health assessments, parental education and anticipatory guidance to prevent both caries progression and its impact on occlusion [11]. Association of early childhood caries and malocclusion patterns: A pedodontic-orthodontic perspective highlighted the significant relationship between early childhood caries and the development of unfavorable occlusal patterns in children. The study emphasized that premature loss and destruction of primary teeth due to caries can contribute to malocclusions such as crowding, crossbite and altered overjet, underlining the importance of early preventive dental care [12]. Timely restorative care and maintenance of primary teeth are essential to preserve arch integrity and prevent space-related discrepancies. When premature loss of primary teeth is unavoidable due to extensive caries, appropriate space management becomes crucial. The use of space maintainers has been widely advocated to prevent mesial drifting of adjacent teeth and subsequent loss of arch length [14]. Without such interventions, children are at a higher risk of developing crowding, ectopic eruption and other orthodontic problems. Interdisciplinary management is a key aspect in addressing the combined impact of ECC and malocclusion. Clinical guidelines highlight the importance of collaboration between pediatric dentists and orthodontists for early identification and interception of developing malocclusions [13, 15]. Such an integrated approach ensures that both disease control and occlusal development are addressed simultaneously. Recent studies have further reinforced the association between ECC severity and the prevalence of malocclusion. Contemporary research indicates that children with higher caries experience tend to exhibit greater incidence of space loss, arch length deficiency and malalignment [2, 16]. These findings support the concept that ECC is not merely a localized dental condition but one that can influence overall craniofacial development. Socioeconomic disparities also play a significant role in shaping both ECC prevalence and malocclusion risk. Limited access to dental care, low parental awareness and unfavorable living conditions contribute to delayed diagnosis and treatment of ECC, thereby increasing the likelihood of complications affecting occlusion [17]. Additionally, inequalities in healthcare access have been shown to influence oral health outcomes and treatment needs in children [18]. Parental knowledge, attitudes and practices are critical determinants in the prevention and management of ECC. Studies have shown that inadequate awareness regarding early oral hygiene practices and feeding behaviors is associated with higher caries prevalence and subsequent orthodontic issues [19]. Improving parental education can therefore play a significant role in reducing both ECC and its long-term sequelae. Furthermore, emerging evidence highlights the importance of early risk assessment and community-based preventive strategies in minimizing the burden of ECC and associated malocclusion. Public health approaches focusing on early screening, fluoride use and dietary counseling can help in reducing disease prevalence and preserving normal occlusal development [20]. Overall, the evidence suggests that ECC and malocclusion share common risk factors and often coexist due to overlapping etiological pathways. Effective management requires a comprehensive approach that integrates preventive, restorative and interceptive orthodontic care. Addressing ECC at an early stage not only improves oral health outcomes but also plays a crucial role in ensuring proper occlusal development and reducing future orthodontic treatment needs.

## Conclusion:

We show a strong association between early childhood caries and specific malocclusion traits, particularly increased overjet, anterior open bite and anterior crossbite. Children with severe ECC showed significantly higher odds of developing these occlusal discrepancies. Behavioral factors such as feeding habits played an important contributory role. Early, integrated pedodontic-orthodontic screening and preventive care are essential to reduce long-term functional and developmental complications.

## Advancement to knowledge:

This study advances current knowledge by demonstrating a clinically relevant association between early childhood caries and the development of malocclusion patterns in children. It highlights how early carious destruction of primary teeth can influence occlusal development, emphasizing the need for an integrated pedodontic-orthodontic approach for early diagnosis and intervention.

## Figures and Tables

**Table 1:** Distribution of ECC severity among children(n = 240)

**ECC Category**	**Number(%)**
No ECC	92(38.3%)
Mild ECC	55(22.9%)
Moderate ECC	25(10.4%)
Severe ECC(S-ECC)	68(28.3%)

**Table 2:** Association between ECC status and malocclusion patterns

**Malocclusion Trait**	**No ECC(%)**	**Mild-ModerateECC(%)**	**Severe ECC(%)**	**p-value**
Increased Overjet	9.5	19.1	31.6	0.017
Anterior Open Bite	8.6	15.4	24.2	0.011
Anterior Crossbite	6.3	9.8	18.4	0.023
Posterior Crossbite	5.4	7.1	13.2	0.082
Crowding	10.8	15.2	23.1	0.067
